# Congruence between Meshes and Library Files of Implant Scanbodies: An In Vitro Study Comparing Five Intraoral Scanners

**DOI:** 10.3390/jcm9072174

**Published:** 2020-07-09

**Authors:** Francesco Mangano, Henriette Lerner, Bidzina Margiani, Ivan Solop, Nadezhda Latuta, Oleg Admakin

**Affiliations:** 1 Private Practice, Gravedona, 22015 Como, Italy; 2 Department of Prevention and Communal Dentistry, Sechenov First Moscow State Medical University, 119991 Moscow, Russia; margiani.b@gmail.com (B.M.); solopivan@yandex.ru (I.S.); latuta.n@mail.ru (N.L.); admakin1966@mail.ru (O.A.); 3 Private Practice, Ludwing-Wilhelm Strasse, 76530 Baden-Baden, Germany; h.lerner@web.de; 4 Academic Teaching and Research Institution of Johann Wolfgang Goethe-University, 60323 Frankfurt am Main, Germany

**Keywords:** Intraoral scanner, Scanbody, Mesh, Library, Congruence, Quantitative evaluation

## Abstract

Purpose. To compare the reliability of five different intraoral scanners (IOSs) in the capture of implant scanbodies (SBs) and to verify the dimensional congruence between the meshes (MEs) of the SBs and the corresponding library file (LF). Methods. A gypsum cast of a fully edentulous maxilla with six implant analogues and SBs screwed on was scanned with five different IOSs (PRIMESCAN^®^, CS 3700^®^, MEDIT i-500^®^, ITERO ELEMENTS 5D^®^, and Emerald S^®^). Ten scans were taken for each IOS. The resulting MEs were imported to reverse engineering software for 3D analysis, consisting of the superimposition of the SB LF onto each SB ME. Then, a quantitative and qualitative evaluation of the deviations between MEs and LF was performed. A careful statistical analysis was performed. Results. PRIMESCAN^®^ showed the highest congruence between SB MEs and LF, with the lowest mean absolute deviation (25.5 ± 5.0 μm), immediately followed by CS 3700^®^ (27.0 ± 4.3 μm); the difference between them was not significant (*p* = 0.1235). PRIMESCAN^®^ showed a significantly higher congruence than MEDIT i-500^®^ (29.8 ± 4.8 μm, *p* < 0.0001), ITERO ELEMENTS 5D^®^ (34.2 ± 9.3 μm, *p* < 0.0001), and Emerald S^®^ (38.3 ± 7.8 μm, *p* < 0.0001). CS 3700^®^ had a significantly higher congruence than MEDIT i-500^®^ (*p* = 0.0004), ITERO ELEMENTS 5D^®^ (*p* < 0.0001), and Emerald S^®^ (*p* < 0.0001). Significant differences were also found between MEDIT i-500^®^ and ITERO ELEMENTS 5D^®^ (*p* < 0.0001), MEDIT i-500^®^ and Emerald S^®^ (*p* < 0.0001), and ITERO ELEMENTS 5D^®^ and Emerald S^®^ (*p* < 0.0001). Significant differences were found among different SBs when scanned with the same IOS. The deviations of the IOSs showed different directions and patterns. With PRIMESCAN^®^, ITERO ELEMENTS 5D^®^, and Emerald S^®^, the MEs were included inside the LF; with CS 3700^®^, the LF was included in the MEs. MEDIT i-500^®^ showed interpolation between the MEs and LF, with no clear direction for the deviation. Conclusions. Statistically different levels of congruence were found between the SB MEs and the corresponding LF when using different IOSs. Significant differences were also found between different SBs when scanned with the same IOS. Finally, the qualitative evaluation revealed different directions and patterns for the five IOSs.

## 1. Introduction

Digital technologies are revolutionising the world of dentistry [1]. The introduction of intraoral scanners (IOSs) [2,3], cone beam computed tomography (CBCT) [4], computer-assisted design and computer-assisted manufacturing (CAD/CAM) software [5], milling machines [6], and three-dimensional (3D) printers [7], together with new highly compatible and aesthetic ceramic materials [8], are transforming workflows in dentistry.

The digital prosthetic workflow is divided into four phases: the acquisition of data with a scanner, which allows obtaining a mesh (ME), i.e., a surface reconstruction of the scanned model; the processing of the ME within CAD software, for designing the prosthetic restorations; the fabrication of the restorations by milling or 3D printing; and finally, the clinical application [1,5,9]. All these steps determine the quality of the clinical result [9,10].

In fixed implant prosthodontics, in particular, the 3D position of the implant is captured through the use of a transfer device, the scanbody (SB), which is screwed on the fixture and scanned with an IOS [11]. This scan, together with that of the master model without SB, the antagonist and the bite are sent in standard tessellation language (STL) format to the dental laboratory, which uses CAD software to model the restorations (individual abutments, temporary, and then definitive restorations) [11]. Within the CAD software, the first and fundamental step performed by the dental technician is the replacement, on the master model, of the ME of the SB with the corresponding library file (LF) [11]. This LF is aligned with all the components (titanium bonding bases with different shape and height) necessary for modelling the prosthetic restorations. Modelling on an LF is certainly preferable to modelling on an ME. The LF, originally designed using CAD software, is geometrically perfect, while the ME is a 3D surface reconstruction that derives from a scan, and is always a geometric approximation of the scanned object; when modelling on an LF, it is possible to obtain perfect marginal adaptation, without any limitation related to the visibility of subgingival structures [11]. The replacement of the ME of the SB with the corresponding LF is possible thanks to the powerful best-fit algorithm of the CAD software, and results in the integration of the LF of the SB, and therefore of the entire library, which is geometrically linked to it, in the master model [11]. From this point, the technician can model all the restorations, which will be fabricated and applied clinically.

Several clinical studies have reported how these protocols can represent a predictable solution for the fabrication of short-span implant-supported restorations (single crowns [11,12,13] and fixed partial prostheses supported by 4–5 implants [6,14,15]). The application of these protocols has a series of advantages, such as the elimination of the conventional impressions with trays and materials, which have always been unwelcome to patients [16], the simplification of clinical procedures, and the saving of time and money, especially when printing physical models is unnecessary [17].

However, several studies [18,19] and literature reviews [20,21] have shown that difficulties persist in fabricating long-span implant-supported restorations (particularly in the case of fixed full arches (FFAs) supported by six or more fixtures) via a full digital workflow, i.e., starting from an optical impression with IOS. These difficulties are mainly attributed to the intrinsic error of IOS, which the literature reports is not sufficiently accurate to capture the impression of multiple implants in the completely edentulous patient [18,19,20,21]. This seems to be mainly related to the mechanism by which the IOS acquires the images, ‘attaching’ frames to each other during the acquisition; therefore, the greater the extent of the scan, the larger the error [19,21]. The intrinsic error of the IOS, however, may not be the only factor determining the inaccuracy. At least four other factors must be considered when capturing an intraoral digital impression: the environment [22], the operator [23], the patient, and the SB [24]. In particular, the SB is still little investigated in the literature [24], but plays a fundamental role in the acquisition. Study of SBs should consider design, material, colour, and tolerances in the fabrication [24,25,26,27]. To date, only a few studies have investigated the influence of these parameters on the quality of the scan [24,25,26,27], and unfortunately, no studies have analysed in depth what happens in the very early stages of CAD modelling, i.e., when the dental technician replaces the SB ME with the corresponding LF. This phase is particularly delicate. If dimensional congruence is not exact between the LF of the SB with the corresponding ME acquired with IOS, problems can arise in the superimposition in CAD, which may result in positional errors [25,27].

Hence, the aim of this in vitro study was to assess and compare the reliability of five different IOSs in the capture of implant SBs, to verify the dimensional congruence between the MEs of the scan abutments captured during the scan of a complete arch model with six implants, and the corresponding LF. The evaluation of the deviations between the MEs of the SBs and the LF was performed using reverse engineering software able to quantitatively and qualitatively assess the incongruences. The null hypothesis was that there was no quantatitive nor qualitative difference between the MEs of the SBs and the LF, and that there were no differences between the different IOSs evaluated.

## 2. Materials and Methods

### 2.1. Study Design

**Figure 1:**
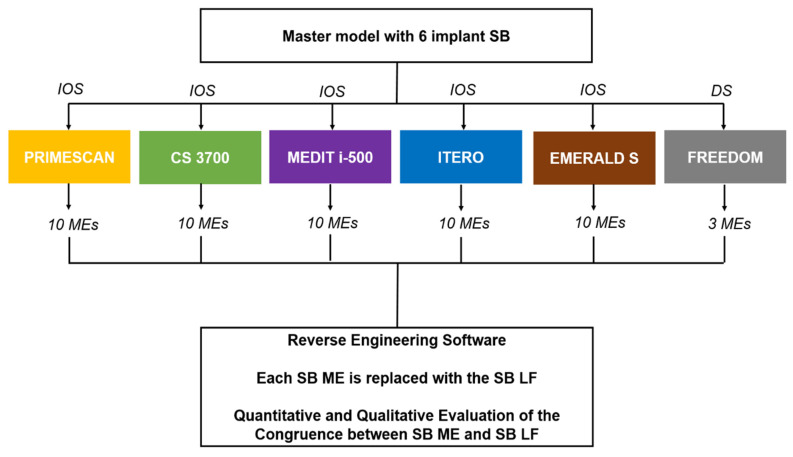
Schematic drawing of the design of the study.

In this study, a gypsum cast representative of a fully edentulous maxilla with six implant analogues and SBs screwed on was scanned with five different IOSs (PRIMESCAN^®^, Dentsply-Sirona, York, PA, USA; CS 3700^®^, Carestream Dental, Atlanta, GA, USA; MEDIT i-500^®^, Medit, Seoul, Korea; ITERO ELEMENTS 5D^®^, Align Technologies, San José, CA, USA; and Emerald S^®^, Planmeca, Helsinki, Finland) and with a desktop scanner (Freedom UHD^®^, Dof Inc., Seoul, Korea). The desktop scans were taken only as a reference and were not included in the comparison. In total, 10 scans were taken for each IOS, for a total of 10 × 5 = 50 MEs, plus 3 desktop scans, for a total of 53 MEs collected. These MEs were saved in specific folders, trimmed to make them uniform and imported to reverse engineering software (Studio^®^, Geomagics, Morrisville, NC, USA) for 3D analysis. The 3D analysis consisted of the superimposition of the SB LF onto each SB ME, using a best-fit algorithm, to replicate the scenario when prosthetic CAD modelling starts. In total, 300 superimpositions were performed for the IOSs plus 18 for the desktop scanner. Then, a quantitative and qualitative evaluation of the deviations between MEs and LF was performed. The results obtained with the different IOSs were evaluated and compared to verify the degree of reliability in the capture of the SB with the different machines. The study design is summarised in Figure 1.

### 2.2. Master Model, SB, and Scanning Procedures

**Figure 2:**
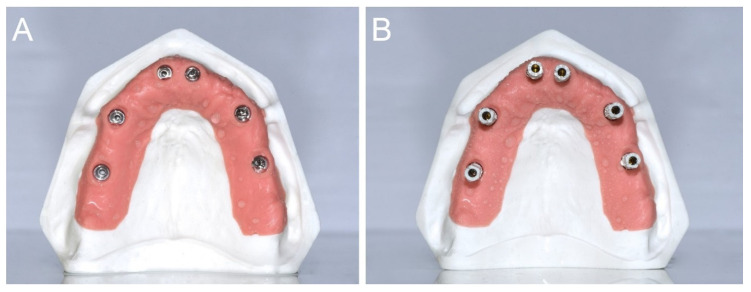
(**A**) A type IV gypsum cast with pink gingiva in the scanabutment area, and the implant analogues in position # 16 (S1), # 14 (S2), # 11 (S3), # 21 (S4), # 24 (S5), and # 26 (S6), respectively, was prepared for the study. (**B**) The gypsum cast with the SBs in position. The SBs were positioned fairly parallel each other.

The gypsum cast was made of type IV plaster with pink gingiva in the scan abutment area, and the implant analogues in positions # 16 (S1), # 14 (S2), # 11 (S3), # 21 (S4), # 24 (S5), and # 26 (S6; Figure 2A). The implant analogues were positioned specularly and not particularly inclined, so that the SBs were positioned fairly parallel to each other (Figure 2B).

**Figure 3:**
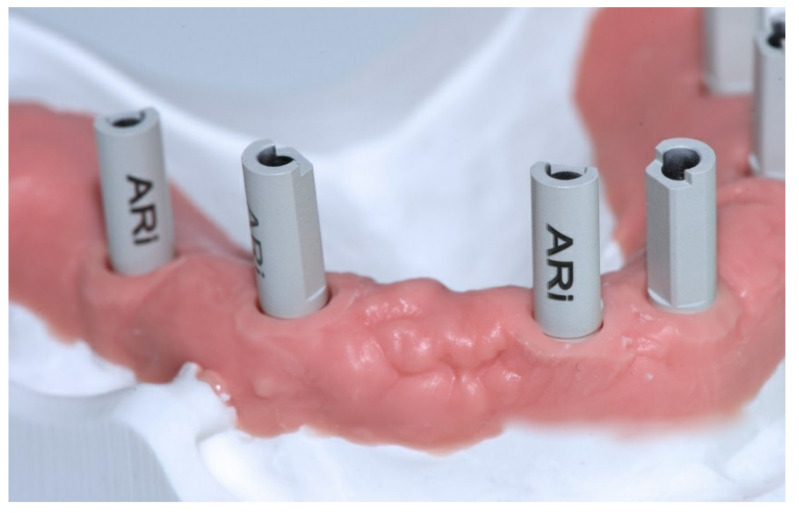
The SBs were all identical, 13 mm in height, and fabricated by the same manufacturer (Megagen, Gyeongbuk, Korea) with the scanning area in opaque white polyether-ether-ketone (PEEK).

The SBs were all identical, 13 mm in height, and fabricated by the same manufacturer (Megagen, Gyeongbuk, Korea) with the scanning area in opaque white polyether ether ketone (PEEK; Figure 3).

The manufacturer reported a maximum tolerance of ±20 μm in the SB production phase. In total, 10 scans were taken per IOS, for a total of 50 MEs captured, by the same implant scanning expert operator (FM). The characteristics of the different IOSs used in this study are summarised in Table 1. For each IOS, the scans were taken using the latests available software version in March 2020.

The IOS scan was limited to the area of the pink gingiva, which was scanned in full, and included capturing the entire SB. To avoid the potential negative effects of operator fatigue, the sequence of scans with the different IOSs was randomised and a 5-min break was scheduled between scans to rest the operator and change the scanner. The scanning strategy used was the same described in a previous study [18]: the zig-zag technique. The operator started from the buccal surface of the model and precisely from the first SB of the right posterior maxilla (# 16), then moved to the occlusal and then palatal side; the operator then returned to the occlusal and then buccal side, moving slowly forward. The progress was slow and constant and the operator tried to capture all the details of the different SBs, without insisting too much on them from the same angle, to avoid excessive reflection. The movement described by the operator was therefore arched, with the scanner head moving over the SB and pink gingiva in a continuous passage from outside to inside, and through a progressive advancing movement. All scans were captured in the same environmental conditions, i.e., in a room with constant temperature (22 °C), controlled humidity (45%) and ambient light, without interference from external light sources. The MEs of the models captured with the different IOSs (10 STL files for each of the 5 IOSs, for a total of 50 MEs) were saved in dedicated folders, labelled with the name of the scanner used. Within each of these folders, the models were numbered from 1 to 10; within each ME, the SBs were numbered from 1 to 6, starting from right posterior area to left. The three desktop scans were taken with an industrial-derived desktop scanner (Freedom UHD^®^, Dof Inc., Seoul, Korea). The Freedom UHD^®^ scanner is a structured light scanner (white light-emitting diode) that acquires the models through two 5.0-megapixel cameras, using patented stable scan stage technology. This technology allows the cameras to move above and around the model to be scanned. It is not the model plate that moves in different positions to facilitate the acquisition of all the details; instead, the lights and cameras move, rotating around the centre of the scan plate, while the model remains stationary. This allows the capture of all the details of the model in a relatively short time (less than 50 s). The scanner has a certified accuracy of 5 μm and generates STL files immediately usable by any CAD. The scanner weighs 15 kg, has dimensions of 330 × 495 × 430 mm, is powered at 110–240 V and 50–60 Hz, and works with Windows operating systems 7, 8 and 10 (64 bit). The three different desktop scans captured were also saved in a dedicated folder as STL files. All the MEs were then cut and trimmed using an individual template to be uniform in size and shape; when uniform, they were saved again in the respective dedicated folders and were ready for analysis.

### 2.3. 3D Analysis of the Congruence between ME and LF

After all scans were captured, each ME was imported into reverse engineering software (Studio^®^, Geomagics, Morrisville, NC, USA), and the LF of the implant SB was superimposed onto the corresponding parts. Six superimpositions were therefore made for each model, for a total of 60 superimpositions per each IOS. Each superimposition entailed two steps. First, the numbered ME, labelled with the name of the IOS used, was loaded into the software; then, six identical SB LFs were loaded, taken directly from the official library of the manufacturer of the implants. These LFs were superimposed, one by one, on the corresponding ME, i.e., on the SB captured by intraoral scanning. The first overlap was by points. The operator (FM) identified three points on each of the SBs present in the MEs acquired with IOS, considering the reference for the overlap; the same points were searched on the SB LF, and the software could thus proceed to a first rough alignment. After this first manual overlap, the operator launched the best-fit algorithm, through which the software perfected the overlaps, one by one, of the SB LFs onto the corresponding SB MEs. The parameters were set with a minimum of 100 iterations per case and the registration used a robust iterative closest point algorithm. With this algorithm, the distances between the SB from ME and library were minimised using a point-to-plane method, and it was possible to calculate the congruence between the structures, expressed quantitatively as the mean ± standard deviation (SD) of the distances between all points of the superimposed models. Finally, for a better qualitative evaluation of the distances between the files and understanding of the directionality of the deviation (i.e., to allow the correct evaluation of the inward and outward deviations), the software allowed generating a colorimetric map. This map was generated through the ‘3D deviation’ function, which made it possible to evaluate the distances between specific points, globally and in all space planes. In this case, the SB LF was considered a reference. Therefore, the colorimetric map indicated inward deviations (defects) with different shades of blue, and outward deviations (excesses) in yellow and red. Minimal deviations were coloured green. The same setting of the colorimetric map was fixed, with the scale ranging from a maximum deviation of +50 to −50 μm, and the best results between +1 and −1 μm (green). The screenshots of the quantitative evaluation and of the colorimetric maps were saved in special folders; particular attention was devoted to capturing screenshots from different angles, to better qualitatively understand on which portion of the SB the major deviations were concentrated. The same process was repeated for the desktop scans.

### 2.4. Outcome Variables

Quantitative deviation between the SB ME and SB LF. This value was calculated with the reverse engineering software, after the application of the best-fit algorithm. It represented the average deviation between the two objects, expressed in mean ± SD, median, range, and 95% confidence interval (CI), in μm.

Qualitative deviation between the SB ME and SB LF. The qualitative deviation was obtained through visual inspection of all samples, using the colorimetric map generated in the software, after the application of the best-fit algorithm. To define this variable, the same experienced operator (FM) who captured all the scans and performed the superimpositions assigned a label of ‘outward deviation’, ‘no deviation’ or ‘inward deviation’, based on the chromatic predominance of red/yellow, green or pale/dark blue, respectively, on each of the three SB portions: flat face central, flat faces lateral, and posterior (back) cylindrical area. This information was collected in a table and expressed as a qualitative variable.

### 2.5. Statistical Analysis

All the data collected were included in datasheets used for statistical analysis. The sample size was determined sufficient for the analysis by a professional statistician. Data analysis and visualisation were performed using R (version 3.6.3) environment for statistical computing (R Foundation for Statistical Computing, Vienna, Austria). For the quantitative evaluation, descriptive statistics for absolute deviations were presented as means (±SD), medians (1st and 3rd quartiles, Q1–Q3), ranges, and 95% CIs. Sample distributions of absolute deviations across different scanners and SBs were visualised using box plots. An observation obtained from S1 SB using the ITERO ELEMENTS 5D^®^ scanner was considered an outlier and not used in the parametric estimation and hypothesis testing procedures. A linear mixed-effects model (implemented in lme4 1.1–21 package) was used to estimate and compare the mean absolute deviations between the IOSs (this model allowed accounting that data have hierarchical properties: scanner → SB). The Tukey method (implemented in emmeans 1.4.5) was used to adjust *p*-values and confidence limits. A linear model (two-way ANOVA with interaction) was used to compare the mean absolute deviations between SBs for each scanner. The Tukey method was used to adjust *p*-values and confidence limits. The Friedman rank test was used to compare models in each type of scanner (SB was considered the blocking variable) with the Holm procedure for multiple testing adjustment. Agglomerative hierarchical biclustering was used to explore scanner–SB relationships regarding averages and variability of the absolute deviations. In order to reduce the α error, the significance level for all tests was established at *p* < 0.01. 

## 3. Results

**Figure 4:**
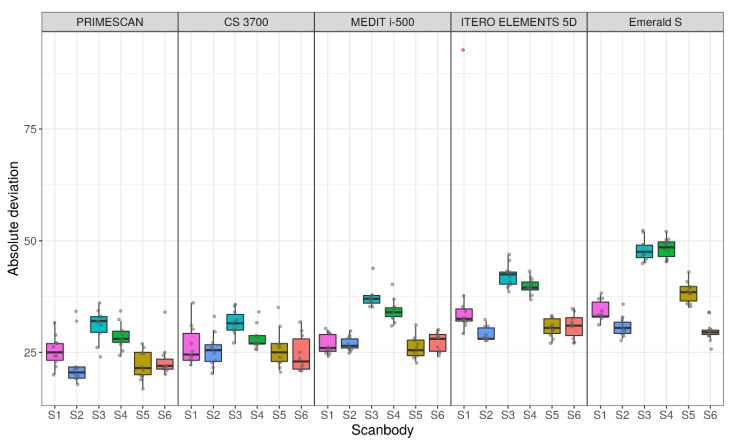
Sample distributions of absolute deviations across IOS and scanbody (SB), in μm.

Descriptive statistics for the absolute deviations in each scanner–SB pair are reported in Figure 4 and Table 2.

**Figure 5:**
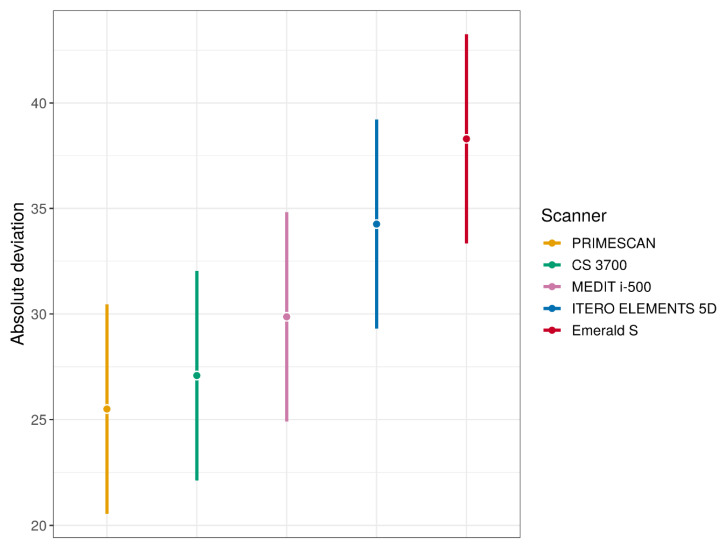
Mean absolute deviations estimates (with 95% confidence interval (CI)) for each type of IOS (these quantities were estimated using linear mixed effects model).

The results of the comparison of the different scanners (estimation and testing using a linear mixed-effects model) are summarised in Figure 5 (means and 95% CIs for each type of scanner) and Table 3 (pairwise differences between means, 95% CIs for differences and *p*-values for pairwise comparisons).

Overall, PRIMESCAN^®^ was the IOS with the lowest mean absolute deviation (25.5 ± 5.0 μm), equal to that of the desktop scanner DOF UHD^®^ (25.5 ± 2.9 μm) used as an external reference in this study. Similar excellent results were also reported for CS 3700^®^ (27.0 ± 4.3 μm), so that the difference between PRIMESCAN^®^ and CS3700^®^ was not statistically significant (*p* = 0.1235). However, the congruence between SB ME and SB LF with PRIMESCAN^®^ was statistically higher than that with MEDIT i-500^®^ (29.8 ± 4.8 μm, *p* < 0.0001), ITERO ELEMENTS 5D^®^ (34.2 ± 9.3 μm, *p* < 0.0001), and Emerald S^®^ (38.3 ± 7.8 μm, *p* < 0.0001). Statistically significant differences were also found when comparing CS 3700^®^ with MEDIT i-500^®^ (*p* = 0.0004), ITERO ELEMENTS 5D^®^ (*p* < 0.0001), and Emerald S^®^ (*p* < 0.0001). Finally, statistically significant differences were found between MEDIT i-500^®^ and ITERO ELEMENTS 5D^®^ (*p* < 0.0001), MEDIT i-500^®^ and Emerald S^®^ (*p* < 0.0001), and ITERO ELEMENTS 5D^®^ and Emerald S^®^ (*p* < 0.0001).

**Figure 6:**
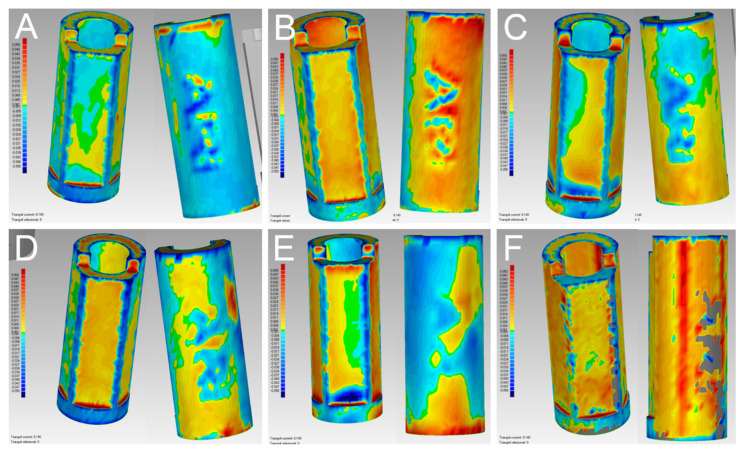
Quantitative evaluation with colorimetric map (frontal and back surface): best results obtained in the study. (**A**) With PRIMESCAN^®^, the best single result amounted to 17 ± 19 μm. (**B**) With CS 3700^®^, the best single result amounted to 20 ± 18 μm. (**C**) With MEDIT i-500^®^ the best single result amounted to 23 ± 26 μm. (**D**) With ITERO ELEMENTS 5D^®^, the best single result amounted to 27 ± 27 μm. (**E**) With Emerald S^®^, the best single result amounted to 26 ± 28 μm. (**F**) With DOF UHD^®^, the best single result amounted to 21 ± 21 μm.

In the quantitative evaluation, among the 60 superimpositions performed in each group, the best single result obtained with PRIMESCAN^®^ was 17 ± 19 μm (Figure 6A), with CS 3700^®^ 20 ± 18 μm (Figure 6B), with MEDIT i-500^®^ 23 ± 26 μm (Figure 6C), with ITERO ELEMENTS 5D^®^ 27 ± 27 μm (Figure 6D), and with Emerald S^®^ 26 ± 28 μm (Figure 6E). The best result obtained with the reference desktop scanner DOF UHD^®^ was 21 ± 21 μm (Figure 6F).

**Figure 7:**
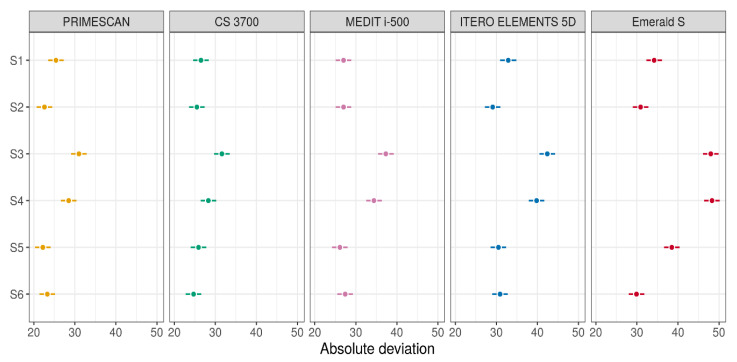
Mean absolute deviations estimates (with 95% confidence intervals) for SBs in each group of IOSs (these quantities were estimated using two-way ANOVA).

The comparison of the deviations between the SBs in each group of IOSs with the results of estimation and testing using the linear model (two-way ANOVA with interaction) with a *p*-value for interaction <0.0001 is summarised in Figure 7 (means and 95% CIs for each type of scanner), Table 4 (pairwise differences between means and 95% CIs for differences), and Table 5 (*p*-values for pairwise comparisons).

With regard to the deviations between the models in each group of scanner, the results of the Friedman test are presented in Table 6. There were no statistically significant differences between the models in each scanner group.

**Figure 8:**
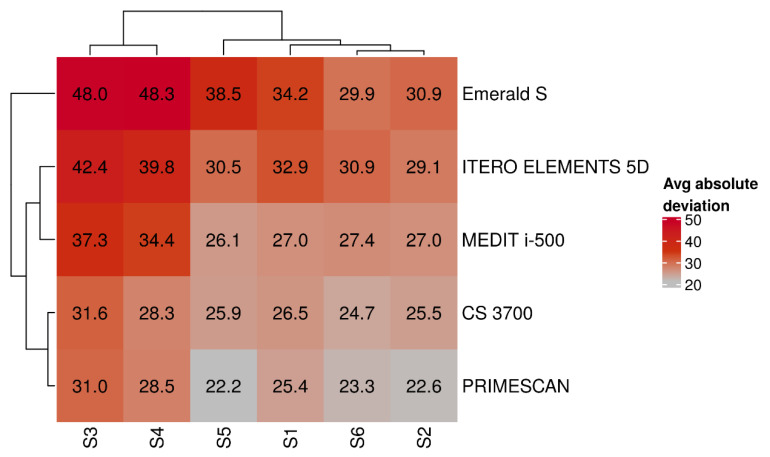
Results of IOSs and SBs biclustering based on average absolute deviation.

**Figure 9:**
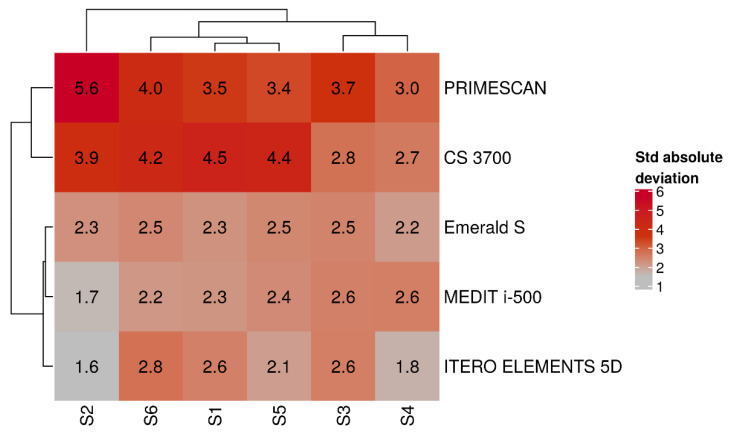
Results of IOSs and SBs biclustering based on standard deviation of absolute deviation.

Hierarchical biclustering results were reported when IOSs and SBs were grouped based on average absolute deviation (Figure 8) and variability (SD) of absolute deviation (Figure 9).

From these latter figures, it was evident that the best results in terms of trueness were obtained by PRIMESCAN^®^ and CS 3700^®^, in correspondence with the SBs in position S2 and S6. Conversely, ITERO ELEMENTS 5D^®^ and MEDIT i-500^®^ revealed the highest repeatability (precision) of the scans with less SD, in correspondence with the SBs in position S4 and S3.

**Figure 10:**
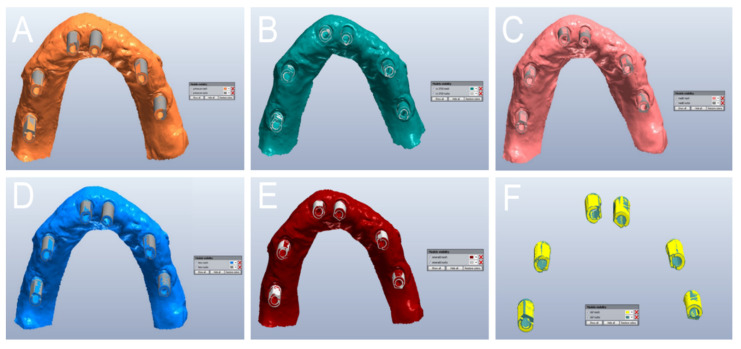
Qualitative evaluation of the deviation patterns (occlusal view of the model). (**A**) With PRIMESCAN^®^, the SB meshes (MEs) were included inside the SB library files (LFs). (**B**) With CS 3700^®^, the SB LFs were included inside the SB MEs. (**C**) With MEDIT i-500^®^, a remarkable interpolation between the SB ME and the SB LF was seen, with no clear direction for the deviation. (**D**) With ITERO ELEMENTS 5D^®^, the SB MEs were included inside the SB LFs. (**E**) With Emerald S^®^, the SB MEs were included inside the SB LFs. (**F**) With DOF UHD^®^, the SB LFs were included inside the SB MEs.

With regard to the qualitative evaluation, the scanners showed different features (Figure 10).

In PRIMESCAN^®^, the SB LF usually included the SB ME; the SB ME did not grow, and appeared to be included within the SB LF (Figure 10A). In contrast, with CS 3700^®^, all the SB MEs included the SB LF, with a marked tendency for the ME to grow, although with minimal quantitative deviations, and in a fairly uniform way (Figure 10B). At the qualitative evaluation, MEDIT i-500^®^ showed a remarkable interpolation between the SB ME and the SB LF, with no clear direction for the deviation (Figure 10C). Finally, ITERO ELEMENTS 5D^®^ (Figure 10D) and Emerald S^®^ (Figure 10E) revealed a pattern and direction of deviation similar to that of PRIMESCAN^®^, with an SB ME that appeared to be included within the SB LF, although with a higher quantitative deviation. DOF UHD^®^ (Figure 10F) showed a deviation pattern similar to that of CS3700^®^.

After visual inspection of all samples, the flat surfaces of almost all SBs showed the best results in terms of deviations (Table 7).

## 4. Discussion

Until now, most studies on the direct digital workflow in implant prosthodontics have focused on the use of IOSs and the intrinsic accuracy of these devices [3,12,18,19,20,21]. The intrinsic error generated during the progression of the intraoral scan has been considered the main reason for the insufficient accuracy of IOSs in taking impressions of completely edentulous patients for the fabrication of implant-supported FFAs [19,20,21]. This intrinsic error exists and certainly plays a role in determining the final inaccuracy of the process, as unequivocally demonstrated by the literature [2,3,12,19,20,21]; however, it is not the only source of error in the full digital workflow in implant prosthodontics.

Other elements contribute to increasing the error: environmental factors (light conditions) [22], factors related to the patient (position, depth and inclination of the implants) [28], factors related to the operator (scanning strategy [23] and experience of the clinician), and finally, the SB [24]. The SB is the transfer that allows capturing the position of the implants in the digital workflow and is therefore crucial. To date, few studies have analysed the influence of factors such as the design of the SB [25,26], the material used to build it [24,26], and the manufacturing tolerances [27] on the error in intraoral scanning. All these elements play a fundamental role and deserve to be adequately investigated by the scientific literature [24].

Even less investigated, however, are the first stages of modelling, in which the dental technician uploads in the CAD software the ME captured by the clinician through intraoral scanning and replaces portions of the SB with the corresponding LF. This moment is key, since an error in this phase can compromise the entire workflow: if a mistake is made at this stage, the individual abutment and prosthetic restoration will be modelled starting from an incorrect implant position [24]. It is therefore important to investigate this phase too, and in particular the congruence between the SB ME and the corresponding LF. Only in the presence of adequate dimensional congruence between these parts can the best-fit algorithm in the CAD software superimpose the files without difficulty, replacing the SB ME with the SB LF [24]. In contrast, in the presence of incongruence between the parts or dimensional deviations, positional errors may arise. Deviations have a detrimental effect when applying the best-fit algorithm, since they can lead to a positional error of the library components on which the dental technician models the prosthetic restorations [24,29]. This may ultimately contribute to a misfit of the prosthetic structure, especially in the case of long-span restorations such as FFAs [24,29].

The purpose of our present in vitro study was therefore to verify the dimensional congruence of MEs of SBs captured with five different IOSs, with the respective LF. This was to quantify the possible error, in micrometres, and to understand not only its presence, but also its direction. For this purpose, a completely edentulous maxilla model was used, with six analogues to which six SBs from the same manufacturer were screwed. This gypsum cast was scanned with five different IOS, and the MEs derived from these scans were loaded into reverse engineering software, where the portions of SBs were aligned to the corresponding LF using the best-fit algorithm. These scans were therefore not considered in their entirety, but the attention was concentrated only on the surface reconstruction of the SB, whose congruence with the corresponding LF was investigated. Therefore, the purpose of the study was not to investigate the general accuracy of the different scanners, which is usually determined by the correct 3D measurement of the distances between the different SBs; rather, the goal was to investigate whether inconsistencies existed between SB MEs and LF, and the presence of any deviations between the files, after the superimposition. To evaluate this, a quantitative and qualitative analysis of the congruence between the files was performed, to quantify the degree of deviation between LF and SB MEs obtained with different IOSs, and to analyse the qualitative characteristics of this deviation, where present. The qualitative analysis, in particular, aimed to establish whether the ME reconstruction of the SB occurred by excess or defect in the various positions, with respect to the reference file of the implant library. The null hypothesis was that there was no quantatitive nor qualitative difference between the MEs of the SBs and the LF, and that there were no differences between the different IOSs evaluated.

At the end of the study, this null hypothesis was rejected. In fact, our present work has highlighted incongruence between SB MEs and SB LF, and that this inconsistency is quantitatively different, with the different scanners. In the present study, the best performance was obtained by PRIMESCAN^®^, which had an average deviation (25.5 ± 5.0 μm) equal to that of the desktop scanner used as an external reference in this study, DOF UHD^®^ (25.5 ± 2.9 μm). The difference between PRIMESCAN^®^ and CS 3700^®^ (27.0 ± 4.3 μm) was minimal and not statistically significant; conversely, MEDIT i-500^®^ (29.8 ± 4.8 μm), ITERO ELEMENTS 5D^®^ (34.2 ± 9.3 μm), and Emerald S^®^ (38.3 ± 7.8 μm) had higher average deviations, and significant differences were found between them, PRIMESCAN^®^ and CS 3700^®^. Finally, significant differences were found also between MEDITi-500^®^, ITERO ELEMENTS 5D^®^ and Emerald S^®^, and between ITERO ELEMENTS 5D^®^ and Emerald S^®^. These differences, measured on numerous samples, can be important in determining the actual accuracy of a CAD project, since this absolute mean error or dimensional discrepancy applies to each SB of the model, and could lead to positional errors.

The qualitative data that emerge from our present in vitro work are also interesting. The error seemed to have a peculiar directionality based on the scanner used. In the case of the desktop scanner and CS 3700^®^, although the average error was quantitatively small, the ME always tended to grow; therefore, the SB LF was always contained within the SB ME, which was larger. With PRIMESCAN^®^, Emerald S^®^, and ITERO ELEMENTS 5D^®^, the opposite happened: the SB ME was contained within the SB LF, at the end of the overlap. This could be linked to post-processing or ‘smoothing’ of the images, through the removal of triangles that exceed the surface reconstruction, by the reconstruction software. The relationship between ME and LF in MEDIT i-500^®^ seems to be more balanced, since there is a sort of interpenetration between the files. More generally, on inspection, the error appears to be less marked on the flat face of the SB; however, this is only qualitative data, because the software used in this study does not allow separately calculating the error present in each of the different faces or geometric parts of the SB. Notably, it is extremely difficult to establish how much these discrepancies can contribute to determining a positional error on the master model that the dental technician uses for modelling. However, having defined their existence, quantified them, and studied their direction are the greatest advantage of our present scientific work.

The CAD best-fit algorithm searches for the congruence between the surfaces of the two STL files of the SB: ME and LF. If congruence is found, superimposition can proceed without errors. If, instead, congruence is not found, i.e., in the case of dimensional differences, the algorithm proceeds using the flat face of the SB as the main reference (if they have more than one, usually the more extended). The flat face undoubtedly represents a valid reference for the best-fit algorithm within the CAD software, but it may also represent an element of dangerous attraction [30]. In case of dimensional differences between the STL files, this face ‘drives’ the superimposition between the files. The result of this process is that the SB LF is ‘dragged’ towards the flat surface of the ME, used as the main reference for the superimposition [30]. This movement can result in a positional error, with a relative shift of the centroids of the objects [30]. This shift, when added up to the intrinsic error given by the intraoral scan, and to the error in milling, may determine the failure or misfit of long-span implant-supported restorations such as FFAs [31].

Although our study is the first to address this issue and is based on the evaluation of a fair number of scans obtained with six different scanners (five IOSs and a desktop scanner), it has limitations. First, it is an in vitro study. Scanning a gypsum cast is certainly easier than in vivo intraoral scanning, which presents technical difficulties due to space limitations, the presence of saliva and possible patient movements [32]. Furthermore, in vitro scanning takes place in light conditions which, although controlled (same environmental light for all IOSs), do not replicate in any way the light conditions of the oral cavity [22]. Since SB reflectance and related ME reconstruction errors vary with light conditions, the data reported in this study need to be critically evaluated. A further limitation of the present study is that the SBs used (six identical SBs produced by the same manufacturer) had not been preliminarily probed with a coordinate measuring machine (CMM) to assess their exact physical dimensions, thus the data relating to manufacturing tolerances were not known. The library is different from the actual SB. In order to make an actual SB, it is produced by an injection method or milling based on the library. These fabrication errors can affect the trueness of the scan: therefore, for the evaluation of the trueness and in order to produce a reference model, reference data for each SB should be obtained using a high-precision CMM machine or industrial 3D optical scanner. Manufacturing tolerances, in fact, play a potentially important role that deserves to be properly investigated, as reported in previous studies [27,33]. However, our present study aimed to investigate the congruence between the SB MEs and the LF, and not the trueness of each SB scan. Not surprisingly, in our study, a statistically significant difference was found between different SBs, in all scanner groups. This could be determined by dimensional differences between the pieces, due to dimensional tolerances given by the production phase. Finally, the strategy used may have favoured some scanners in the correct reconstruction of ME, penalising others; certainly, there is a link between the scanner acquisition technology and the scanning strategy, although the literature has not adequately clarified this [34]. Further in vivo studies are therefore necessary to obtain more information, to better define the influence of the first CAD phases on the final error in the full digital workflow in implant prosthodontics. These studies also need to be extended to other implant systems.

## 5. Conclusions

Our present in vitro study aimed to compare the reliability of five different IOSs (PRIMESCAN^®^, CS 3700^®^, MEDIT i-500^®^, ITERO ELEMENTS 5D^®^, and Emerald S^®^) in the capture of implant SBs and to verify the dimensional congruence between the MEs of the SBs and the corresponding LF. At the end of the quantitative evaluation, statistically different levels of congruence were found between the SB MEs captured with the different IOSs and the corresponding LF. PRIMESCAN^®^ and CS 3700^®^ showed the highest congruence between SB MEs and LF, with the lowest mean absolute deviations (25.5 ± 5.0 μm and 27.0 ± 4.3 μm, respectively); the difference between these two scanners and the other three was statistically significant. Significant differences were also found between MEDIT i-500^®^ and ITERO ELEMENTS 5D^®^, MEDIT i-500^®^ and Emerald S^®^, and ITERO ELEMENTS 5D^®^ and Emerald S^®^. Based on these results, the null hypothesis for this study was rejected, since deviations were found between the MEs of the SBs and the LF, and significant differences were found among the different IOSs evaluated. Significant differences were also found among different SBs, but no differences were found between the models scanned with the same IOS. Finally, the qualitative evaluation revealed different directions and patterns for the five IOSs investigated. Further studies are needed to confirm these preliminary results.

## Figures and Tables

**Table 1:** Features of the different intraoral scanners (IOSs) used in this study.

Name	Producer	Technology	Colour	Output
PRIMESCAN^®^	Dentsply-Sirona	High-resolution sensors and shortwave light with optical high-frequency contrast analysis for dynamic deep scan (20 mm)	yes	dxd (proprietary format) and stl (open format) with Connect
CS 3700^®^	Carestream Dental	Active triangulation with smart-shade matching via bidirectional reflectance distribution function	yes	dcm (proprietary format); ply and stl (open formats)
MEDIT i-500^®^	Medit	3D in motion video technology	yes	obj, ply and stl (open formats)
ITERO ELEMENTS 5D^®^	Align Technologies	Parallel confocal microscopy	yes	3ds (proprietary format); ply and stl (open formats)
Emerald S^®^	Planmeca	Projected pattern triangulation	yes	ply and stl (open formats)

**Table 2:** Descriptive statistics: mean (SD); median; (Q1–Q3), in μm.

Scanner	S1	S2	S3	S4	S5	S6
PRIMESCAN^®^	25.4 (3.5); 25.0; (23.2–27.0)	22.6 (5.6); 20.5; (19.2–21.8)	31.0 (3.7); 32.0; (29.5–33.0)	28.5 (3.0); 28.0; (27.2–29.8)	22.2 (3.4); 21.5; (20.0–25.0)	23.3 (4.0); 22.0; (21.2–23.5)
CS 3700^®^	26.5 (4.5); 24.5; (23.2–29.2)	25.5 (3.9); 25.5; (23.0–26.8)	31.6 (2.8); 31.5; (30.0–33.5)	28.3 (2.7); 27.0; (27.0–28.8)	25.9 (4.4); 25.0; (23.0–27.0)	24.7 (4.2); 23.0; (21.2–28.0)
MEDIT i-500^®^	27.0 (2.3); 26.0; (25.2–29.0)	27.0 (1.7); 26.5; (26.0–28.0)	37.3 (2.6); 37.0; (36.0–37.8)	34.4 (2.6); 34.0; (33.0–35.0)	26.1 (2.4); 25.5; (24.2–27.8)	27.4 (2.2); 28.0; (25.2–29.0)
ITERO ELEMENTS 5D^®^	38.9 (19.2); 32.5; (32.0–34.8)	29.1 (1.6); 28.0; (28.0–30.5)	42.4 (2.6); 42.5; (40.2–43.0)	39.8 (1.8); 39.5; (39.0–40.8)	30.5 (2.1); 30.5; (29.2–32.5)	30.9 (2.8); 31.0; (28.8–32.8)
Emerald S^®^	34.2 (2.3); 33.0; (33.0–36.2)	30.9 (2.3); 30.5; (29.2–31.8)	48.0 (2.5); 47.5; (46.2–49.0)	48.3 (2.2); 48.5; (46.5–49.8)	38.5 (2.5); 38.5; (36.5–39.8)	29.9 (2.5); 29.5; (29.0–30.0)

**Table 3:** Comparisons of mean absolute deviations between the IOS.

Contrast (Pairwise Comparisons)	Difference	95% CI for Difference	*p*
PRIMESCAN^®^—CS 3700^®^	−1.58	[−3.41; 0.24]	0.1235
PRIMESCAN^®^—MEDIT i-500^®^	−4.37	[−6.19; −2.54]	<0.0001
PRIMESCAN^®^—ITERO ELEMENTS 5D^®^	−8.76	[−10.59; −6.92]	<0.0001
PRIMESCAN^®^—Emerald S^®^	−12.80	[−14.63; −10.97]	<0.0001
CS 3700^®^—MEDIT i-500^®^	−2.78	[−4.61; −0.96]	0.0004
CS 3700^®^—ITERO ELEMENTS 5D^®^	−7.17	[−9.01; −5.34]	<0.0001
CS 3700^®^—Emerald S^®^	−11.22	[−13.04; −9.39]	<0.0001
MEDIT i-500^®^—ITERO ELEMENTS 5D^®^	−4.39	[−6.22; −2.56]	<0.0001
MEDIT i-500^®^—Emerald S^®^	−8.43	[−10.26; −6.61]	<0.0001
ITERO ELEMENTS 5D^®^—Emerald S^®^	−4.04	[−5.88; −2.21]	<0.0001

**Table 4:** Comparisons of mean absolute deviations between SBs in each group of IOSs (mean differences and 95% CIs for them).

	PRIMESCAN^®^	CS 3700^®^	MEDIT i-500^®^	ITERO ELEMENTS 5D^®^	Emerald S^®^
S6 − S5	1.10 [−2.81; 5.01]	−1.20 [−5.11; 2.71]	1.30 [−2.61; 5.21]	0.40 [−3.51; 4.31]	−8.60 [−12.51; −4.69]
S6 − S4	−5.20 [−9.11; −1.29]	−3.60 [−7.51; 0.31]	−7.00 [−10.91; −3.09]	−8.90 [−12.81; −4.99]	−18.40 [−22.31; −14.49]
S6 − S3	−7.70 [−11.61; −3.79]	−6.90 [−10.81; −2.99]	−9.90 [−13.81; −5.99]	−11.50 [−15.41; −7.59]	−18.10 [−22.01; −14.19]
S6 − S2	0.70 [−3.21; 4.61]	−0.80 [−4.71; 3.11]	0.40 [−3.51; 4.31]	1.80 [−2.11; 5.71]	−1.00 [−4.91; 2.91]
S6 − S1	−2.10 [−6.01; 1.81]	−1.80 [−5.71; 2.11]	0.40 [−3.51; 4.31]	−1.99 [−6.01; 2.03]	−4.30 [−8.21; −0.39]
S5 − S4	−6.30 [−10.21; −2.39]	−2.40 [−6.31; 1.51]	−8.30 [−12.21; −4.39]	−9.30 [−13.21; −5.39]	−9.80 [−13.71; −5.89]
S5 − S3	−8.80 [−12.71; −4.89]	−5.70 [−9.61; −1.79]	−11.20 [−15.11; −7.29]	−11.90 [−15.81; −7.99]	−9.50 [−13.41; −5.59]
S5 − S2	−0.40 [−4.31; 3.51]	0.40 [−3.51; 4.31]	−0.90 [−4.81; 3.01]	1.40 [−2.51; 5.31]	7.60 [3.69; 11.51]
S5 − S1	−3.20 [−7.11; 0.71]	−0.60 [−4.51; 3.31]	−0.90 [−4.81; 3.01]	−2.39 [−6.41; 1.63]	4.30 [0.39; 8.21]
S4 − S3	−2.50 [−6.41; 1.41]	−3.30 [−7.21; 0.61]	−2.90 [−6.81; 1.01]	−2.60 [−6.51; 1.31]	0.30 [−3.61; 4.21]
S4 − S2	5.90 [1.99; 9.81]	2.80 [−1.11; 6.71]	7.40 [3.49; 11.31]	10.70 [6.79; 14.61]	17.40 [13.49; 21.31]
S4 − S1	3.10 [−0.81; 7.01]	1.80 [−2.11; 5.71]	7.40 [3.49; 11.31]	6.91 [2.89; 10.93]	14.10 [10.19; 18.01]
S3 − S2	8.40 [4.49; 12.31]	6.10 [2.19; 10.01]	10.30 [6.39; 14.21]	13.30 [9.39; 17.21]	17.10 [13.19; 21.01]
S3 − S1	5.60 [1.69; 9.51]	5.10 [1.19; 9.01]	10.30 [6.39; 14.21]	9.51 [5.49; 13.53]	13.80 [9.89; 17.71]
S2 − S1	−2.80 [−6.71; 1.11]	−1.00 [−4.91; 2.91]	0.00 [−3.91; 3.91]	−3.79 [−7.81; 0.23]	−3.30 [−7.21; 0.61]

**Table 5:** Comparisons of mean absolute deviations between SBs in each group of scanners (*p*-values).

	PRIMESCAN^®^	CS 3700^®^	MEDIT i-500^®^	ITERO ELEMENTS 5D^®^	Emerald S^®^
S6 − S5	0.9661	0.9509	0.9318	0.9997	<0.0001
S6 − S4	0.0023	0.0910	<0.0001	<0.0001	<0.0001
S6 − S3	<0.0001	<0.0001	<0.0001	<0.0001	<0.0001
S6 − S2	0.9956	0.9918	0.9997	0.7734	0.9776
S6 − S1	0.6382	0.7734	0.9997	0.7147	0.0219
S5 − S4	0.0001	0.4929	<0.0001	<0.0001	<0.0001
S5 − S3	<0.0001	0.0006	<0.0001	<0.0001	<0.0001
S5 − S2	0.9997	0.9997	0.9860	0.9085	<0.0001
S5 − S1	0.1789	0.9979	0.9860	0.5290	0.0219
S4 − S3	0.4455	0.1526	0.2762	0.3997	0.9999
S4 − S2	0.0003	0.3147	<0.0001	<0.0001	<0.0001
S4 − S1	0.2082	0.7734	<0.0001	<0.0001	<0.0001
S3 − S2	<0.0001	0.0002	<0.0001	<0.0001	<0.0001
S3 − S1	0.0007	0.0030	<0.0001	<0.0001	<0.0001
S2 − S1	0.3147	0.9776	1.0000	0.0774	0.1526

**Table 6:** Comparisons between models (M) in each group of scanners (row p-values and adjusted for multiple comparisons).

Scanner	*p*	p_adj_
PRIMESCAN^®^	0.0147	0.0737
CS 3700^®^	0.3971	1.0000
MEDIT i-500^®^	0.3879	1.0000
ITERO ELEMENTS 5D^®^	0.3452	1.0000
Emerald S^®^	0.2700	1.0000

**Table 7:** Distribution of the deviations in the different surfaces of the SBs, by visual inspection.

Scanner	Deviation	Flat Central	Flat Lateral	Back
PRIMESCAN^®^	Outward deviation	+	+	+
	No deviation	++	++	++
	Inward deviation	++	+++	+++
CS 3700^®^	Outward deviation	+++	+++	+++
	No deviation	++	+	+
	Inward deviation	+	+	+
MEDIT i-500^®^	Outward deviation	++	++	++
	No deviation	++	+	+
	Inward deviation	++	++	++
ITERO ELEMENTS 5D^®^	Outward deviation	+	+	+
	No deviation	++	+	+
	Inward deviation	+++	+++	+++
Emerald S^®^	Outward deviation	+	+	+
	No deviation	+	+	+
	Inward deviation	+++	+++	+++
